# Resequencing of two elite sorghum (*Sorghum bicolor* (L.) Moench) hybrid parent lines reveals distinctly different genome-wide variation models

**DOI:** 10.1038/s41598-025-18320-3

**Published:** 2025-09-26

**Authors:** Xing-Long Li, Fang-Chao Gao, Fei Li, Sha Tang, Ji-Hong Li, Zhen-Yu Zhao, Yu-Bin Chen, Xing-Yu Chen, Zhi Wang, Qingpo Liu, Gui-Ying Li

**Affiliations:** 1 College of Advanced Agricultural Sciences, Zhejiang A&F University, Lin’an, Hangzhou, 311300 China; 2 Institute of Crop Sciences, Chinese Academy of Agricultural Sciences, Beijing, 100081 China; 3 Crop Resources Institute of Jilin Academy of Agricultural Sciences, Gongzhuling, 130033 Jilin China; 4 Kweichow Moutai Distillery (Group) Hongyingzi Science & Technology Development Co., Ltd, Renhuai, 564512 Guizhou China

**Keywords:** *Sorghum bicolor*, Heterosis, AJ2055, RN133, Genome-wide variation, Plant breeding, Plant genetics

## Abstract

Sorghum (*Sorghum bicolor* (L.) Moench), the world’s fifth most economically important cereal crop, is renowned for its exceptional drought tolerance and water-use efficiency. Hybrid breeding, a cornerstone strategy for yield enhancement through the exploitation of heterosis, has been extensively employed in sorghum improvement. Despite its agricultural importance, the molecular mechanisms driving heterosis remain largely unresolved. To address this knowledge gap, we conducted whole-genome resequencing of two elite parental lines, AJ2055 and RN133, which have contributed to the development of over 30 commercial hybrids. Our objectives were to delineate genomic variations associated with heterosis performance and elucidate their functional implications in yield-related traits. Genomic analyses revealed substantial divergence between the two parental lines: AJ2055 harbored 2,961,777 single nucleotide polymorphisms (SNPs) and 474,247 insertions and deletions (InDels), whereas RN133 exhibited 54,724 structural variations (SVs) and 36,515 copy number variations (CNVs). Chromosomes 5 and 10 showed the highest SNP densities, with distinct spatial distribution patterns distinguishing the two lines. Notably, RN133 exhibited a broader and more diverse array of genetic variations compared to AJ2055, potentially underlying its superior performance in hybrid combinations. Functional annotation of the variant genes revealed their involvement in essential metabolic pathways, including carbon metabolism, starch and sucrose metabolism, and hormone signaling-related pathways. Key genes encoding enzymes such as phosphoenolpyruvate carboxylase, 1,4-alpha-glucan-branching enzyme, and cytochrome P450 were found to harbor non-synonymous mutations, potentially influencing yield-related traits and stress adaptation. These findings provide valuable insights into the genetic basis of heterosis and lay a foundation for the development of molecular markers for sorghum breeding. In conclusion, this study highlights the distinct patterns of genomic variation between AJ2055 and RN133 and their potential candidate genes and pathways involved in heterosis. The identification of yield-related genes and pathways offers a theoretical basis for future research on sorghum genetic improvement and hybrid breeding. Through the integration of multi-omics approaches and advanced breeding technologies, these findings will contribute to the development of high-yielding sorghum varieties.

**Supplementary Information:**

The online version contains supplementary material available at 10.1038/s41598-025-18320-3.

## Introduction

Sorghum (*Sorghum bicolor* (L.) Moench) is the world’s fifth most important cereal crop, following wheat, rice, maize, and barley^1^. As a C4 crop, sorghum exhibits high levels of drought resistance and salinity tolerance, requires relatively little water, and demonstrates water-use efficiency, making it a vital crop in arid and semi-arid regions^2^. In China, sorghum is an indispensable feedstock for the famous Chinese Baijiu (Chinese liquor) making^3^.

Heterosis, or hybrid vigor, refers to the phenomenon in which hybrid offsprings outperform their parental lines in traits such as growth, yield, and stress resistance. It is a crucial genetic phenomenon widely exploited in modern crop breeding^4^. Since Shull proposed the concept of heterosis in the early twentieth century, its potential in crop breeding has attracted considerable attention^5^. The utilization of heterosis has significantly enhanced the yield and quality of many crops, with hybrid varieties of major staple crops such as maize, rice, and sorghum now widely adopted globally^6,7^. In sorghum breeding, a wide range of male sterile (A) lines and restorer (R) lines have been developed in China. Among them, the sterile line AJ2055 and the restorer line RN133 have been widely utilized in hybrid breeding. For instance, AJ2055 has served as the female parent in 33 hybrid combinations, such as Jiza 210 (AJ2055/RN133), Jiza 130 (AJ2055/0-30), and Deza 9 (AJ2055/Y16)^8^. Similarly, RN133 has been used in six hybrids, such as Siza 25 (TAM428/RN133), Jiza 97 (352A/RN133)^9^, and Jinza 33 (SX605A/RN133)^10^, among others.

The genetic basis of heterosis involves multiple levels, including gene expression regulation, allelic interactions, structural variations (SVs), and single nucleotide polymorphisms (SNPs)^11,12^. For instance, structural variations play important roles in the formation of heterosis, particularly in rice, where they significantly contribute to genetic diversity and hybrid vigor^11^. Additionally, gene expression regulation and allelic interactions are widely recognized as key drivers of heterotic effects^13,14^. Studies suggest that the manifestation of heterosis is closely related to the complementarity between parental genomes, which can be predicted using molecular marker technologies^15,16^.

With the release of the whole genome sequence of sorghum cultivar BTx623 as the reference genome for sorghum^17^, genomic research in sorghum has advanced substantially, and genetic variation is now being studied in a more comprehensive manner. Such genetic variation has been analyzed at the whole-genome level, including single nucleotide polymorphisms (SNPs), small insertions and deletions (InDels), structural variations (SVs), copy number variations (CNVs), and presence/absence variants (PAVs)^18–20^. The identification of a large number of genetic variations in the sorghum genome, alongside more accurate and comprehensive variation data obtained from the pan-genome, has provided valuable genetic resources for genetic improvement of sorghum. Several yield-related QTLs and genes have been identified^21^. For example, Boyles et al. identified several significant SNP loci associated with grain number per panicle, grain weight per panicle, and thousand grain weight^22^. Similarly, Zhao et al. identified 101 SNP loci associated with at least one of the nine traits through GWAS analysis of cereal and biomass-related plant morphological traits in sorghum. Among these, the GA biosynthesis gene KS3, located at a major locus on chromosome 6, is strongly associated with seed number^23^.

In this study, two elite sorghum hybrid parents, AJ2055 and RN133, were resequenced using whole-genome resequencing technology and compared with the published BTx623 reference genome to identify their sequence polymorphisms and structural variation patterns. This work identified a large number of SNPs, InDels, SVs, and CNVs. Analysis of these variants revealed potential genomic regions involved in starch and carbon metabolism that are likely associated with yield. Overall, this study provides important insights for the genetic improvement of sorghum through crossbreeding.

## Results

### Genome-wide identification of genetic variations

**Fig. 1:**
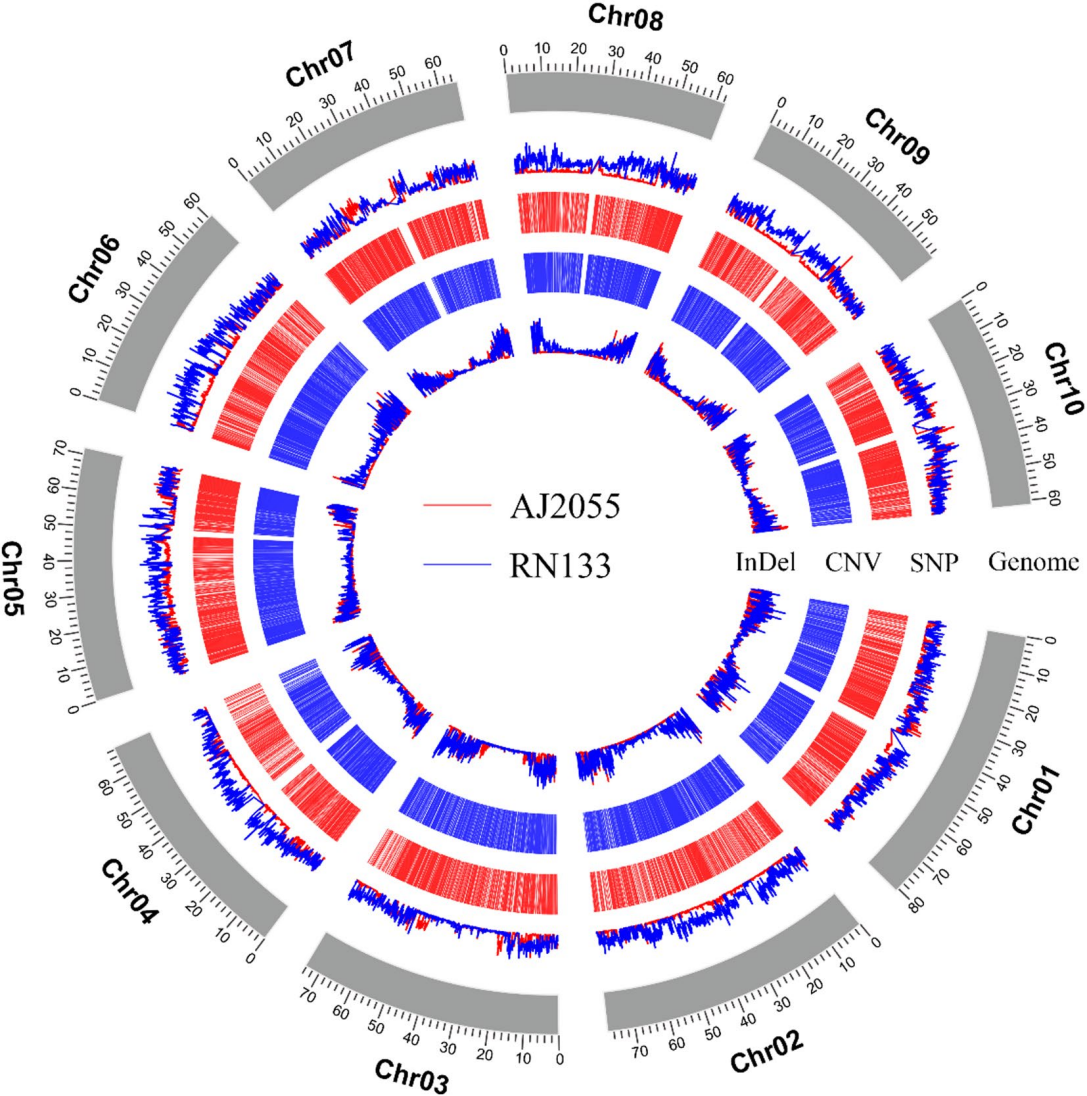
Genome-wide landscape of the variations in AJ2055 and RN133. The outermost rings represent the chromosome. The second rings show single nucleotide polymorphism (SNP) of AJ2055 (red) and RN133 (blue). The third and fourth rings represents copy number variation (CNV) duplication and deletion of AJ2055 and RN133. The fifth rings illustrate insertions and deletion (indel) of AJ2055 (red) and RN133 (blue).

**Fig. 2:**
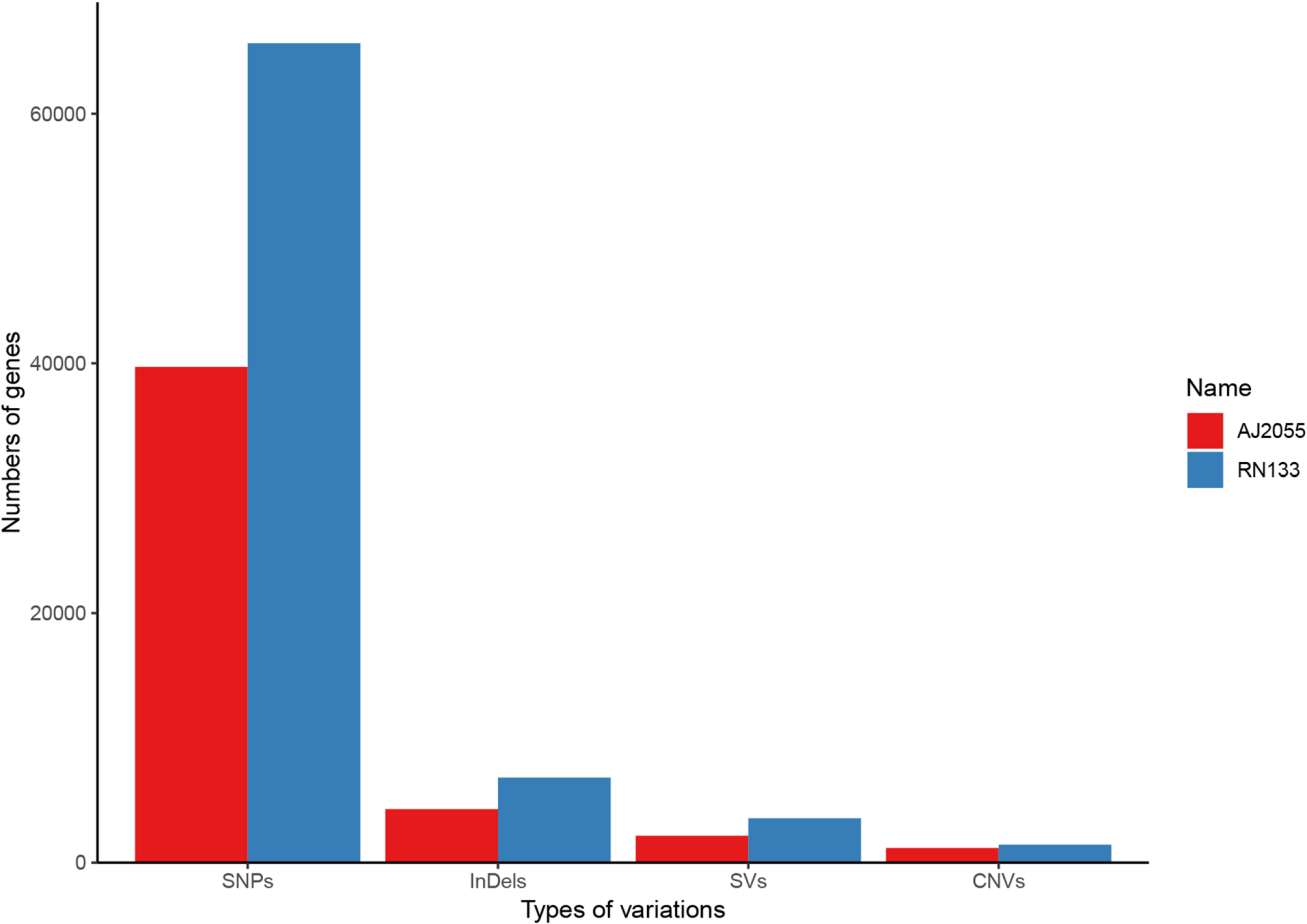
A summary of the number of genes across the four types of genetic variations.

We completed the whole-genome sequencing of the sorghum varieties AJ2055 and RN133, generating a total of 79.61 Gb of raw resequencing data, including 79.27 Gb of high-quality, filtered reads (Table 1; NCBI accessions SRR32375442 and SRR32375443). The average sequencing depth for the two sorghum varieties was 45 × , with coverage depths ranging from 42.56 × to 47.44 × . This resequencing depth is adequate for genetic variation analysis. Using high-quality sequencing data aligned to the BTx623 reference genome, we identified a large number of SNPs, InDels, SVs, and CNVs in AJ2055 and RN133. A total of 2,961,777 SNPs were detected, including 1,029,447 from AJ2055 and 1,932,330 from RN133; 474,247 InDels (177,391 from AJ2055 and 296,856 from RN133); 54,724 SVs (21,110 from AJ2055 and 33,614 from RN133); and 36,515 CNVs (15,710 from AJ2055 and 20,805 from RN133) (Figs. 1, 2, Supplementary Fig. S1). The distribution of these variations across the 10 chromosomes was uneven, with chromosome 10 exhibiting the most variation in AJ2055, while chromosome 5 exhibited the highest variation in RN133. A small subset of variations was localized to specific chromosomes. For each individual, different types of variants were clustered on different chromosomes. For example, AJ2055 had the most SNPs on chromosome 10, the most InDels on chromosome 1, and the most CNVs on chromosome 5. In contrast, RN133 showed the highest number of SNPs and CNVs on chromosome 5 and the most InDels on chromosome 1.

**Table 1:** Summary of sequencing data quality.

Name	Raw base (Gb)	Clean base(Gb)	Effective rate (%)	Error rate (%)	Q20 (%)	Q30 (%)	GC content (%)
AJ2055	37.38	37.18	99.42	0.03	97.53	93.17	43.75
RN133	42.23	42.09	99.7	0.03	96.81	91.21	43.73

According to the annotation, RN133 had the highest number of SNPs in the upstream, exon, intronic, and downstream regions. Meanwhile, the heterozygosity rate of both varieties was less than 50%, indicating a relatively low level (Table 2). Subsequently, a total of 4,291 InDels were identified in the coding region of AJ2055, including 2,229 deletions, 2,032 insertions, and 30 InDels that introduced a premature stop codon. In RN133, a total of 6,818 InDels were identified in the coding region, including 3,424 deletions, 3335 insertions, 55 InDels that introduced a premature stop codon, and 4 InDels that resulted in the loss of a stop codon (Fig. 2, Supplementary Table S1).

**Table 2:** Statistics of SNP variant gene regions and mutation types.

Name	Upstream	Stop gain	Stop loss	Synonymous	Non-synonymous	Intronic	Downstream	Heterogenous rate	Total
AJ2055	46,998	214	92	19,948	19,334	70,669	37,125	0.306	1,029,447
RN133	78,048	389	122	33,344	31,623	122,010	62,658	0.454	1,932,330

We also analyzed the SVs and CNVs in two sorghum varieties. Annotation revealed that in the exonic coding regions, AJ2055 contained 2,157 SVs and 1175 CNVs, whereas RN133 harbored 3,558 SVs and 1,443 CNVs (Fig. 2, Supplementary Table S1).

### Comparison of non-synonymous mutations of SNPs and InDels among the two parent lines

**Fig. 3:**
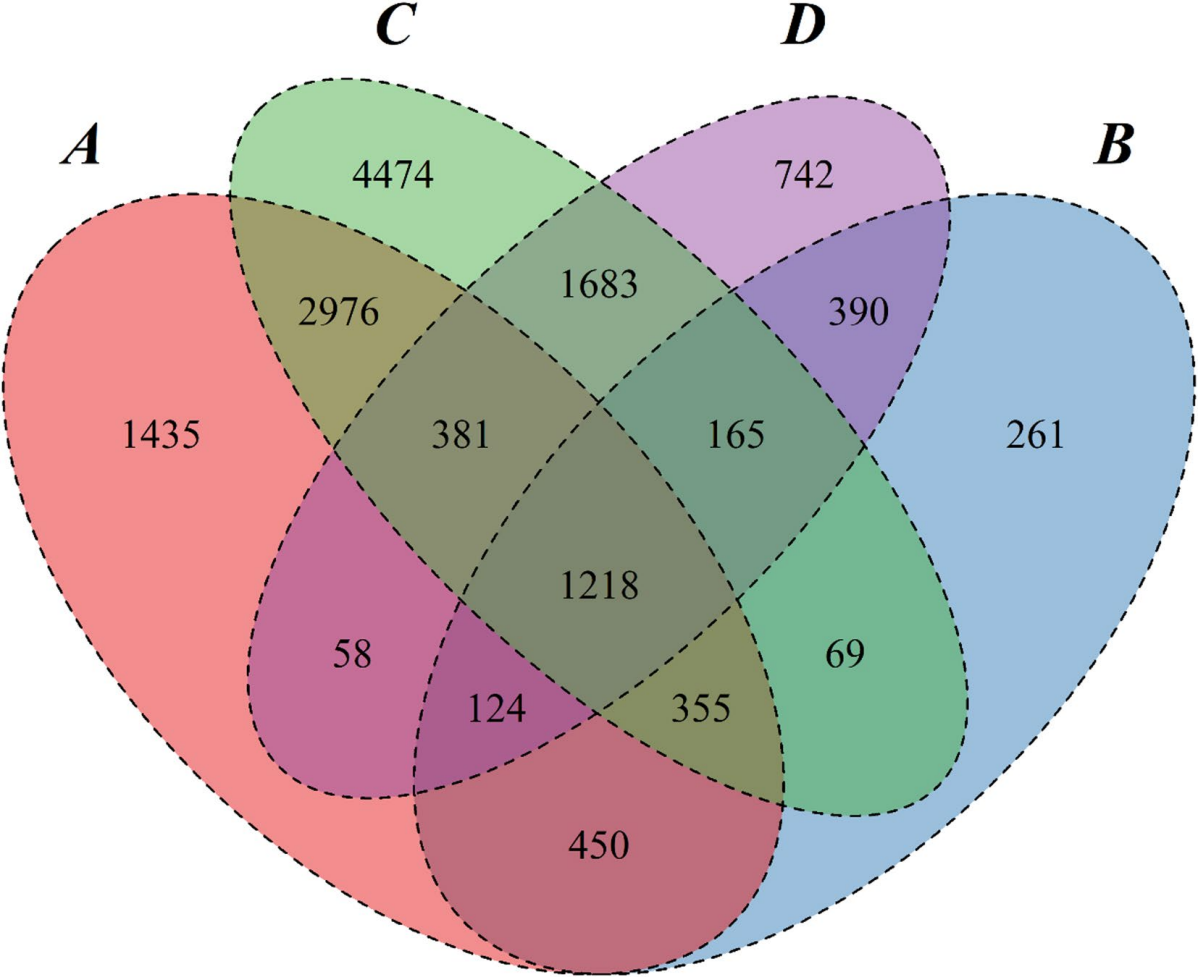
Gene Venn diagram of non-synonymous mutations of SNPs and InDels of the 2 sorghum lines. **(A)** SNPs variant gene of AJ2055. **(B)** InDels variant gene of AJ2055. **(C)** SNPs locus variant gene of RN133. **(D)** InDels variant gene of RN133.

The Venn diagram (Fig. 3) clearly illustrates the commonalities and differences in the variation sites between the two sorghum varieties. Specifically, there are 4,930 shared SNP variation sites between AJ2055 and RN133, accounting for 70.46% of AJ2055’s and 43.55% of RN133’s total SNP variation sites, respectively. The intersection of InDel variation sites is 1,897, representing 62.57% of AJ2055’s and 39.84% of RN133’s total InDel variation sites, respectively. Furthermore, the overlap between SNPs and InDels in AJ2055 is 2,147, while that in RN133 is 3,447. Additionally, AJ2055 exhibits 2,067 (29.54%) unique SNP variation sites compared to RN133, while RN133 displays 6,391 (56.45%) unique SNP variation sites. For InDel variation sites, AJ2055 has 1,135 (37.43%) unique sites, while RN133 has 2,864 (60.61%) unique InDel variation sites. These results demonstrate significant shared genetic variation alongside distinct differences in genetic variation between the two sorghum varieties. Although a large proportion of the variation sites are shared, the presence of unique SNPs and InDels in AJ2055 and RN133 highlights the genomic distinctiveness of these two varieties.

### Enrichment of functional genes with genetic variation

**Fig. 4:**
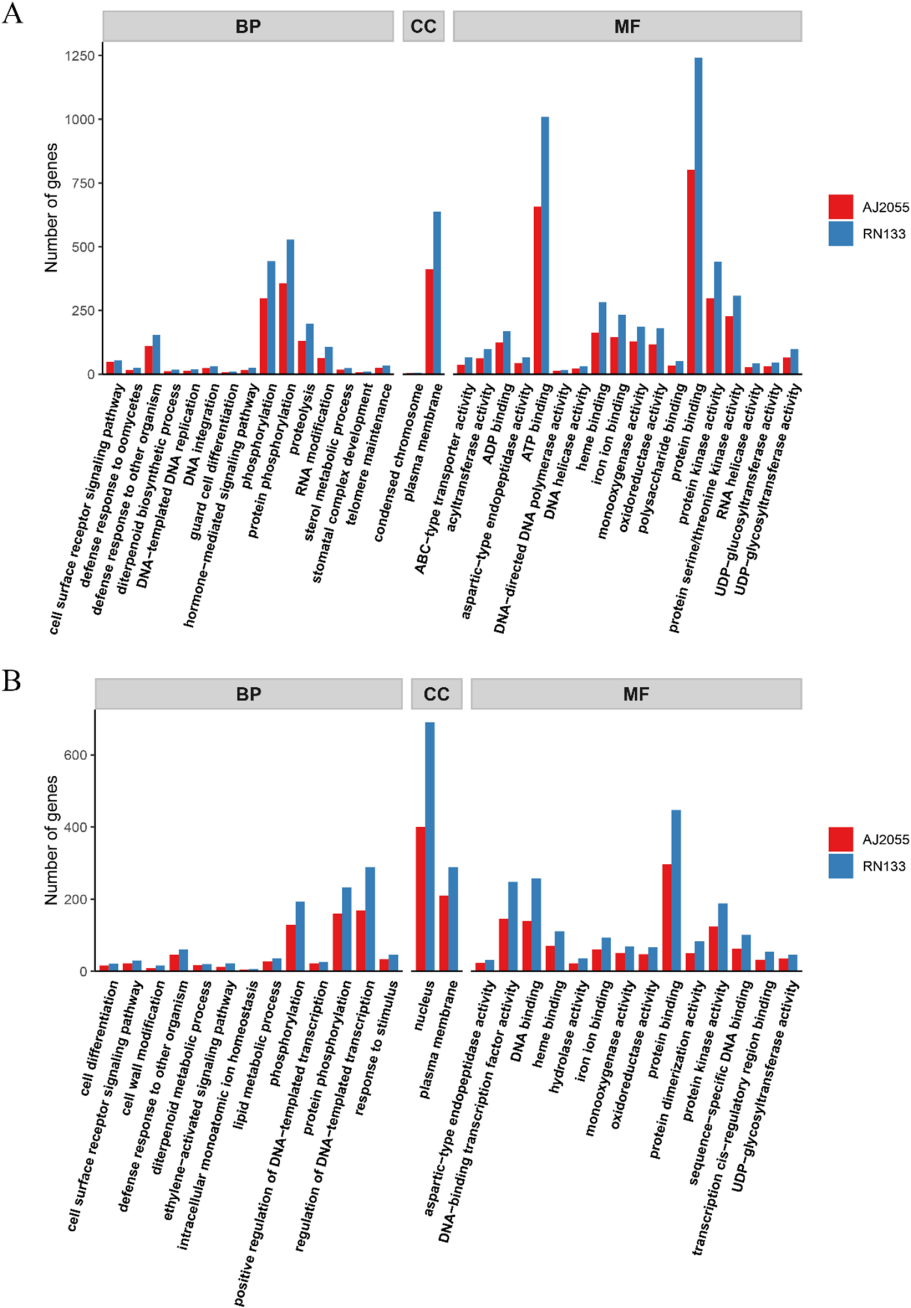
GO enrichment analysis. **(A)** and **(B)** Go enrichment analysis of SNPs and InDels variant in two sorghum varieties (*p* < 0.05). Red represents AJ2055, and blue represents RN133. The analysis includes three categories: Biological Process (BP), Cellular Component (CC) and Molecular Function (MF).

The analysis was conducted on the SNP and InDel variants present in two sorghum varieties. The results of the Gene Ontology (GO) annotation revealed that the SNP and InDel variants shared by the two sorghum cultivars (AJ2055 and RN133) were distributed across three GO ontologies (Fig. 4). For both SNPs and InDels, the number of variants was higher in RN133 than in AJ2055. Among the biological process’s ontologies, protein phosphorylation and phosphorylation-related genes were the most abundant in the SNP variants of the two varieties, accounting for 7.44% and 6.19% in AJ2055, and 7.46% and 6.26% in RN133, respectively (Fig. 4A). Among the InDel variants, regulation of DNA-templated transcription and protein phosphorylation were the most common biological process, accounting for 6.77% and 6.41% in AJ2055, and 7.43% and 5.99% in RN133, respectively (Fig. 4B). In the cellular component ontology, the genes with the most SNP variants were concentrated in the plasma membrane, accounting for 8.56% in AJ2055, and 9.02% in RN133. Additionally, the unique SNPs and InDels of the two sorghum varieties were analyzed, and the results showed that RN133 had more GO enrichments than AJ2055. The most abundant GO category in AJ2055 was membrane-related, whereas the InDel-based analysis revealed that RN133 contained more membrane-related genes (Supplementary Fig. S1).

**Fig. 5:**
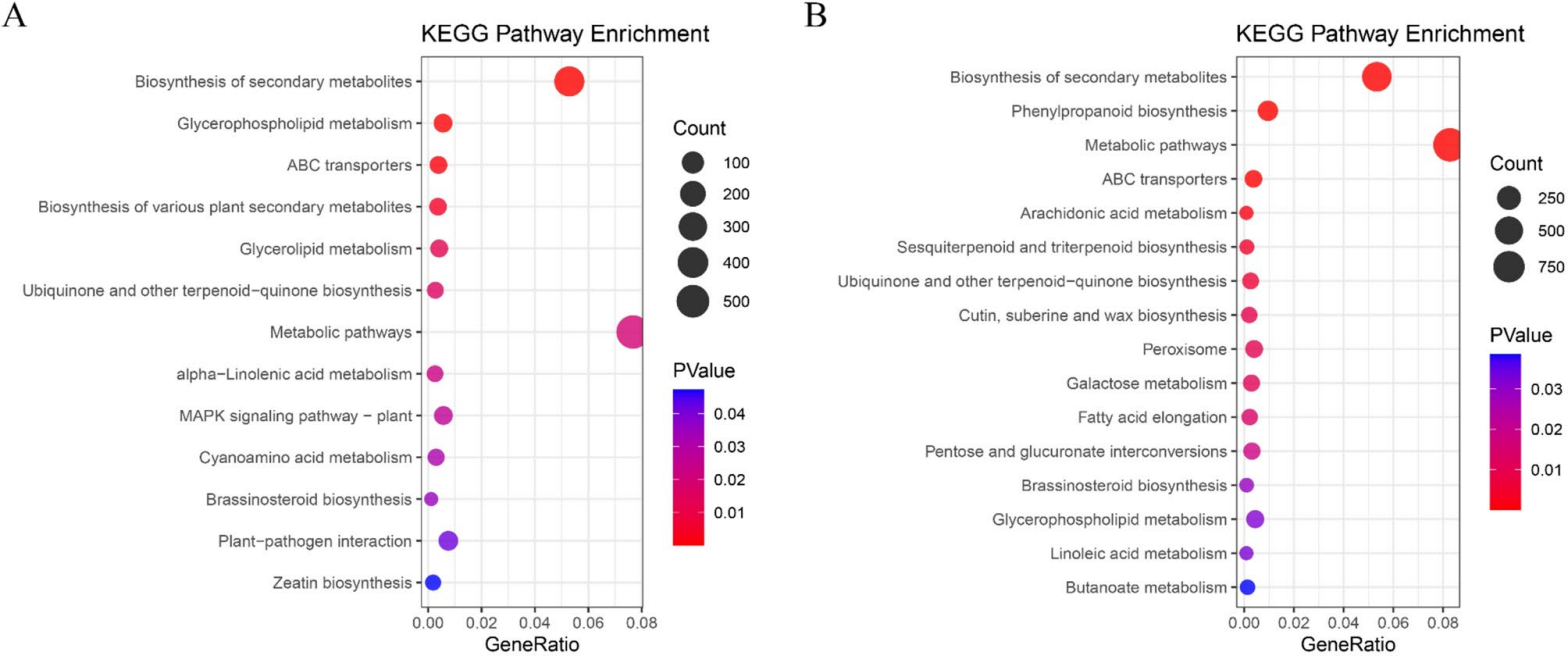
Significant enrichment pathway of SNPs variants. **(A)** and **(B)** KEGG enrichment analysis of SNPs variants in two sorghum varieties (*p* < 0.05). Red represents AJ2055, and blue represents RN133.

Among the InDel variants, the majority of variant genes were located in the nucleus, accounting for 16.02% in AJ2055 and 17.76% in RN133. Among the molecular function ontologies, protein binding and ATP binding were the most abundant functional categories in the SNP variants of AJ2055 and RN133, with proportions of 16.71% and 13.69% in AJ2055, and 17.55% and 14.26% in RN133, respectively. For the InDel variants, protein binding accounted for the highest proportion in both cultivars, at 11.89% in AJ2055 and 11.48% in RN133. In the KEGG pathway annotation, 537 and 937 SNP variants in AJ2055 and RN133, respectively, were involved in metabolic pathways (Fig. 5). In addition, 37 InDel variants in AJ2055 were associated with plant hormone signal transduction, while 202 InDel variants in RN133 were associated with the biosynthesis of secondary metabolites (Supplementary Fig. S1).

We further applied a false discovery rate (FDR) correction to the enrichment results. For SNP variations, significantly enriched pathways included the common pathways ABC transporters and Biosynthesis of secondary metabolites, as well as the AJ2005-specific pathway Glycerophospholipid metabolism, and the RN133-specific pathways Arachidonic acid metabolism, Metabolic pathways, and Phenylpropanoid biosynthesis (Supplementary Fig. S1). For InDel variations, significantly enriched pathways included the common pathway Plant hormone signal transduction, the AJ2005-specific pathway MAPK signaling pathway–plant, and the RN133-specific pathway Plant–pathogen interaction (Supplementary Fig. S1).

### Variation of genes associated with yield

**Fig. 6:**
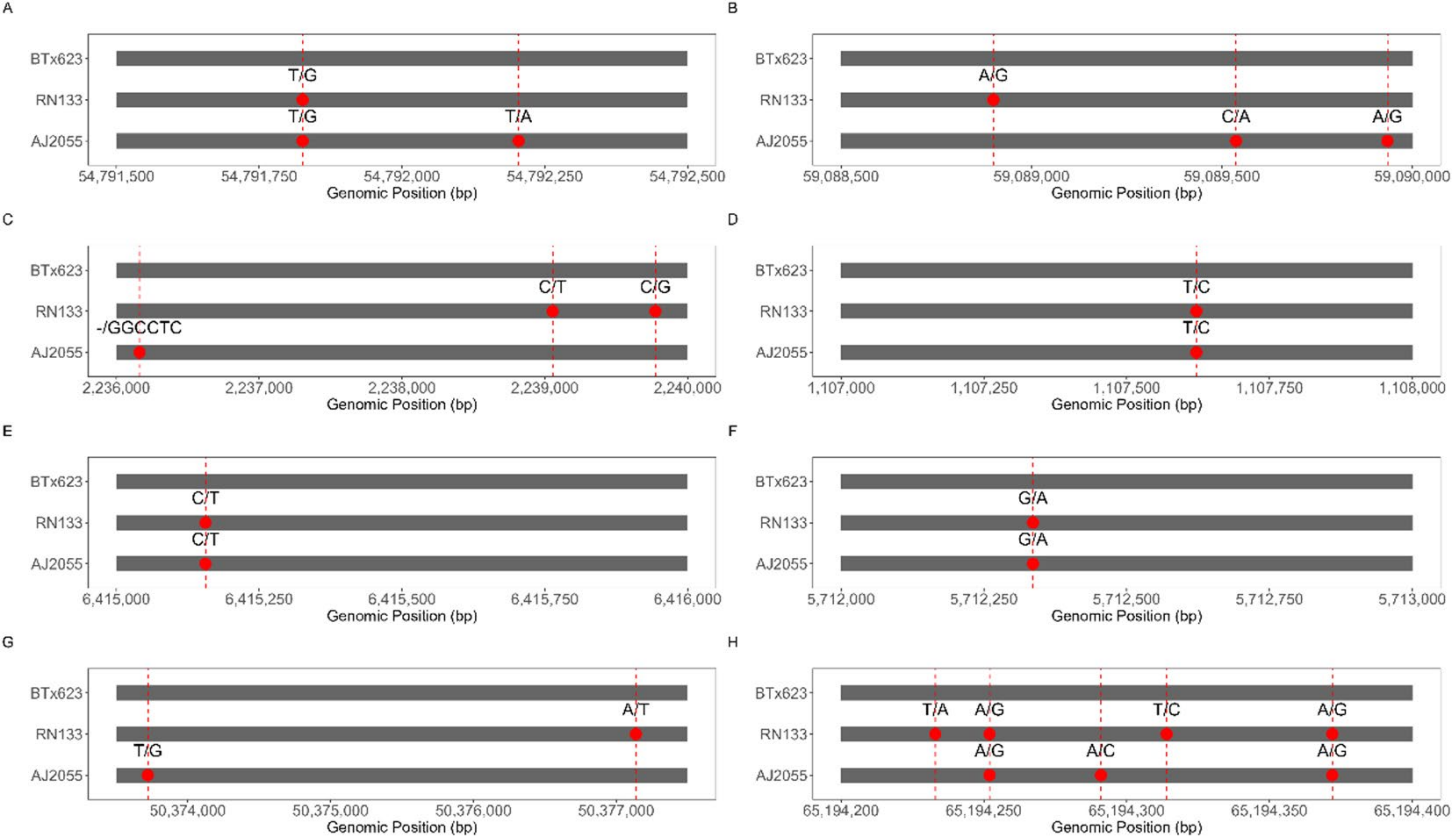
Comparison of DNA sequence variation sites for 8 nonsynonymous mutation genes. **(A)** SORBI_3003G213800. **(B)** SORBI_3007G156400. **(C)** SORBI_3004G027800. **(D)** SORBI_3004G013500. **(E)** SORBI_3004G078000. **(F)** SORBI_3005G056400. **(G)** -/8055248. **(H)** SORBI_3002G216500.

In comparison with the reference genome BTx623, we found 46, 48 and 61 variant genes involved in carbon metabolism, starch and sucrose metabolism, and hormone-related metabolism, respectively, in AJ2055, and 82, 84 and 11 in RN133 (Supplementary Table S1). Among these, phosphoenolpyruvate carboxylase, D-glycerate 3-kinase, fructose-bisphosphate aldolase, glyoxylate/succinic semialdehyde reductase 2, phosphoglycerate kinase, and ribulose-phosphate 3-epimerase are involved in photosynthesis and the Calvin cycle, and genetic variants in these genes are present in both sorghum cultivars (Table 3). Notably, the phosphoenolpyruvate carboxylase gene is present in both cultivars with the same haplotype. Similarly, mutations in the 1,4-alpha-glucan-branching enzyme 3, 4-alpha-glucanotransferase DPE1, alpha-amylase, alpha-amylase isozyme, beta-amylase, and soluble starch synthase 2-3 genes were found in both AJ2055 and RN133. The proteins encoded by these genes are involved in starch biosynthesis originating from the Calvin cycle (Table 3). In addition, shared variants in cytochrome P450 71A1 and cytochrome P450 90A4 are involved in brassinolide synthesis. Eight key genes associated with non-synonymous mutations were identified. These genes are involved in essential biological processes, including carbohydrate metabolism, energy metabolism, secondary metabolism, and response to environmental stimuli. A detailed comparison of their DNA sequence variation sites was conducted, providing a comprehensive overview of the genetic differences (Fig. 6). Among them, gene A encodes a 1,4-α-glucan branching enzyme, which regulates the formation of starch branched chains. Genes B and C encode enzymes that catalyze starch degradation via α-amylase and β-amylase, respectively. Gene D is involved in UDP-glucose synthesis and contributes cell wall metabolism. Gene E functions as citrate synthase, driving the tricarboxylic acid cycle. Genes F and G regulate carbon fixation and ATP generation through the Calvin cycle and photosynthesis, respectively, while gene H encodes cytochrome P450 71A1, which mediates secondary metabolite synthesis to enhance stress resistance. Variations in these genes may influence plant energy balance, growth and development, and stress adaptation by modifying protein function or regulatory networks, thereby offering potential molecular targets for crop quality enhancement and stress resistance breeding.

**Table 3:** Functional annotation of variant genes associated with carbon metabolism and starch/sucrose pathways.

Gene ID/ Entrez Gene ID	Gene description	Function
SORBI_3004G106900	phosphoenolpyruvate carboxylase 1	Catalyze phosphoenolpyruvate and carbon dioxide to form oxaloacetic acid
SORBI_3010G160700	phosphoenolpyruvate carboxylase 3	Catalyze phosphoenolpyruvate and carbon dioxide to form oxaloacetic acid
SORBI_3003G259200	D-glycerate 3-kinase, chloroplastic	Catalyze the formation of D-glycerate 3-phosphate
SORBI_3005G056400	fructose-bisphosphate aldolase, chloroplastic	The condensation of dihydroxyacetone phosphate and glyceraldehyde phosphate produces fructose-1, 6-diphosphate
SORBI_3003G195150	glyoxylate/succinic semialdehyde reductase 2, chloroplastic	Catalyze the reduction of glyoxylic acid and hydroxypyruvate
N/A-/(8,055,248)	phosphoglycerate kinase, chloroplastic	Catalytic reduction of 1, 3-diphosphoglyceric acid to 3-phosphoglyceric acid
SORBI_3001G491000	ribulose-phosphate 3-epimerase, chloroplastic	Catalyzed isomerization of ribulose 5-phosphate
SORBI_3003G213800	1,4-alpha-glucan-branching enzyme 3, chloroplastic/amyloplastic	Branching the glucose chain by forming alpha-1, 6-glucoside bonds increases the solubility and availability of starch
SORBI_3002G383700	4-alpha-glucanotransferase DPE1, chloroplastic/amyloplastic	Transfer glucose residues and regulate the structure of starch
SORBI_3003G276400	alpha-amylase 3, chloroplastic	Catalyze the hydrolysis of starch
SORBI_3007G156400	alpha-amylase isozyme 3B	Similar function of starch hydrolysis
SORBI_3002G225600	alpha-amylase isozyme 3C	Similar function of starch hydrolysis
SORBI_3001G293800	beta-amylase 3, chloroplastic	Similar function of starch hydrolysis
SORBI_3002G329400	beta-amylase	Similar function of starch hydrolysis
SORBI_3002G329500	beta-amylase	Similar function of starch hydrolysis
SORBI_3004G027800	beta-amylase 8	Similar function of starch hydrolysis
SORBI_3010G093400	soluble starch synthase 2-3, chloroplastic/amyloplastic	Catalyze glucose-1-phosphate to form starch
SORBI_3002G216500	cytochrome P450 71A1	Participate in secondary metabolism
SORBI_3005G030400	cytochrome P450 90A4	Biosynthesis of the plant hormone brassinolide

## Discussion

Following the release of the reference genome BTx623^17^ and the rapid advancement of high-throughput sequencing and bioinformatics tools^24,25^, an increasing number of sorghum germplasm resources have been resequenced^18,26^, enabling deeper insights into genetic variation. In this study, we characterized genetic variation between sterile and restorer lines, providing valuable resources for sorghum hybrid breeding and molecular marker-assisted selection.

In this study, high-throughput sequencing technology was used to analyze the genetic variation between two elite sorghum parents: the sterile line AJ2055 and the restorer line RN133. Compared with the BTx623 reference genome sequence, a large number of variant sites were identified between the two parents, including 2.96 million SNPs, 470,000 InDels, 50,000 SVs, and 30,000 CNVs. The number of breeding combinations involving the restorer parent RN133 was found to exceed that of the sterile line parent AJ2055, and the genetic variation in RN133 was determined to be more abundant. The genomic differences between AJ2055 and RN133 were the result of multiple interacting factors, including breeding objectives, germplasm background, and varietal adaptability. Specifically, the sterile line AJ2055 was developed through successive backcrossing between an improved maintainer line and an original sterile line, whereas the restorer line RN133 was bred using a multi-parent hybridization strategy, ultimately yielding a genetically stable line. This breeding approach not only endowed RN133 with enhanced environmental adaptability but also led to the accumulation of more genetic variation.

In the functional annotation of genetic variants, the intergenic regions of AJ2055 and RN133 accounted for 76.15% and 78.72% of the SNP variants, and 56.91% and 58.21% of the InDel variants, respectively. Although these non-coding regions do not encode proteins, they are enriched with cis-regulatory elements such as enhancers and silencers that modulate gene expression through interactions with transcription factors^27–29^. Among the coding regions, 3-bp InDel variants were the most prevalent, as they do not induce frameshift mutations and typically result in the insertion or deletion of a single amino acid. Due to their relatively mild impact on protein function, such mutations may be preferentially retained by natural selection, contributing moderate variation to functional evolution^18,30^.

Functional annotation in this study focused primarily on non-synonymous mutations within coding regions. GO annotation and KEGG pathway enrichment revealed that AJ2055 and RN133 shared a high degree of similarity in variant genes and yield-related metabolic pathways, suggesting that these patterns of functional divergence are not randomly distributed but rather reflect directional selection or adaptive evolution^31^. In contrast, distinct enrichment patterns were detected between the two cultivars. AJ2055 showed significant enrichment in the glycerophospholipid metabolism pathway, which may influence pollen mother cell membrane stability via altered lipid composition, thereby contributing to the male sterility phenotype through lipid peroxidation^32^. RN133, on the other hand, was significantly enriched in the phenylpropanoid biosynthesis pathway, whose metabolic products may act synergistically to enhance drought resistance^33,34^. These genomic differences directly influence the formation of heterosis. By comparing the distribution of genetic variations and gene functions between the two cultivars, it revealed that the parental genomes may drive hybrid vigor through complementary or over dominant effects. This phenomenon has been previously observed in the superior yield traits of Jiza 210 ^35,36^. Currently reported RF genes are located on sorghum chromosomes 2, 4, 5, 7, and 8. The higher genetic variations observed in RN133 compared to AJ2055 across these chromosomal regions suggest that these variations could contribute to the formation of novel allelic variants in RN133, thereby enhancing its restoring ability^37^.

Despite significant progress in heterosis research, several challenges remain in its practical application. For instance, the expression of heterosis is highly influenced by environmental conditions, and further studies are needed to ensure its stability across diverse environments^38^. Additionally, the underlying genetic mechanisms are complex, involving multiple genes and regulatory networks, thus necessitating deeper investigation to enable more precise breeding strategies^39,40^. In the future, the integration of multi-omics technologies and the development of intelligent breeding platforms are expected to enable more systematic and precise crop improvement, providing critical support for global food security and sustainable agricultural development^41,42^. In recent years, intelligent breeding strategies powered by artificial intelligence and big data analytics have propelled heterosis research into a new era^43,44^. The application of molecular marker-assisted selection (MAS) and genome-wide association studies (GWAS) has led to enhanced prediction efficiency and breeding accuracy of heterosis^45–47^. Moreover, the genomic selection (GS) models provide new insights into the genetic basis of heterosis^48,49^.

## Conclusions

In this study, we performed whole-genome resequencing of two elite sorghum parent lines, AJ2055 and RN133, to explore genomic variation underlying heterosis. RN133 exhibited greater genetic variation, especially in structural variants and copy number variations, which may contribute to its superior performance in hybrid combinations. Variants were unevenly distributed across chromosomes, and functional annotation revealed enrichment in carbon metabolism, starch and sucrose metabolism, and hormone-related pathways, all closely tied to yield traits.

Furthermore, non-synonymous mutations in key genes involved in development and stress response suggest functional roles in heterosis expression. The complementary genetic landscapes of AJ2055 and RN133 provide a valuable resource for molecular marker-assisted selection and heterosis optimization in sorghum breeding.

## Materials and methods

### Plant materials and genome sequencing

The plant materials used in this study, AJ2055 and RN133, were developed by the Crop Resources Institute of Jilin Academy of Agricultural Sciences and Siping Academy of Agricultural Sciences, Jilin Province, respectively, and were provided by the Institute of Crop Science, Chinese Academy of Agricultural Sciences. Plants were cultivated in 2023 at the Changping Experimental Station of the Institute of Crop Science. Genomic DNA was extracted from young leaves using the CTAB method^50^. Following quality assessment, the genomic DNA was randomly fragmented into 350 bp segments using a Covaris ultrasonic. Library construction involved end repair, 3’-end poly(A) tailing, adapter ligation, purification, and polymerase chain reaction amplification. The resulting libraries were subjected to paired-end (PE150) sequencing on the Illumina NovaSeq 6000 sequencing platform by Beijing Novogene Technology Co., Ltd (Beijing, China).

## Read filtering and mapping

The original sequencing data were subjected to quality control based on the following filtering criteria: (1) Reads containing adaptors were removed; (2) When the N content of the single-ended sequencing read exceeds 10% of the length ratio of the read, the corresponding paired-end reads were removed. (3) When the number of low-quality (Q <  = 5) bases in the single-ended sequencing reads exceeds 50% of the length ratio of the read, the corresponding paired-end reads were removed. Filtered valid reads were then aligned to the reference genome using BWA v0.7.17 (mem-t4-k32-M) and duplicates reads were removed using SAMtools v1.13 (rmdup). The BTx623 reference genome sequence was downloaded from the NCBI https://www.ncbi.nlm.nih.gov/datasets/genome/GCF_000003195.3.

## Detection and annotation of variations

Both SNPs and InDels were identified using SAMtools v1.13 (mpileup-m2-F 0.002-d1000-u-C50). Variants supported by fewer than four reads or with a mapping quality (MQ) score below 20 were filtered out to accurate detection. SVs were identified using BreakDancer v1.4.4 (-q 20), which detects insertions, deletions, inversions, intra-chromosomal translocations, and inter-chromosomal translocations, based on paired-end read alignments and insert size deviations relative to the reference genome. SVs supported by fewer than two paired-end reads were filter out. CNVs were detected using CNVnator v0.3 (-call 100), which is based on read depth analysis to identify potential deletions and duplications across the genome. All identified variants were annotated using ANNOVAR.

## Gene variation analysis

The reference genome BTx623 was used as the reference control. Genes harboring non-synonymous SNPs and InDels in the coding regions of AJ2055 and RN133 were selected for analysis. These genes were converted to Entrez Gene IDs using the Gene ID Conversion Tool available on the DAVID website (https://david.ncifcrf.gov/home.jsp), and subsequently subjected to Gene Ontology (GO) and Kyoto Encyclopedia of Genes and Genomes (KEGG) enrichment analysis. Enrichment significance was evaluated using the hypergeometric distribution test, and significantly enriched pathways (*p* < 0.05)-particularly those related to carbon metabolism and starch and sucrose metabolism, were identified using Fisher’s exact test. Multiple testing correction was performed using the false discovery rate (FDR) method, and pathways with an FDR-adjusted *p* value < 0.05 were considered significantly enriched.

## Supplementary Information

Below is the link to the electronic supplementary material.

Supplementary Material 1

Supplementary Material 2

## Data Availability

The original datasets generated during the current study are available in the National Center for Biotechnology Information (NCBI) BioProject repository, PRJNA1224864.
