# Supplementary material for: Resequencing of two elite sorghum (Sorghum bicolor (L.) Moench) hybrid parent lines reveals distinctly different genome-wide variation models
Source: Sci Rep. 2025 Sep 26;15:33125. doi: 10.1038/s41598-025-18320-3 (PMC12475106; doi:10.1038/s41598-025-18320-3)
Supplement: Supplementary file 2 — Supplementary Material 2 [file 41598_2025_18320_MOESM2_ESM.docx]

**
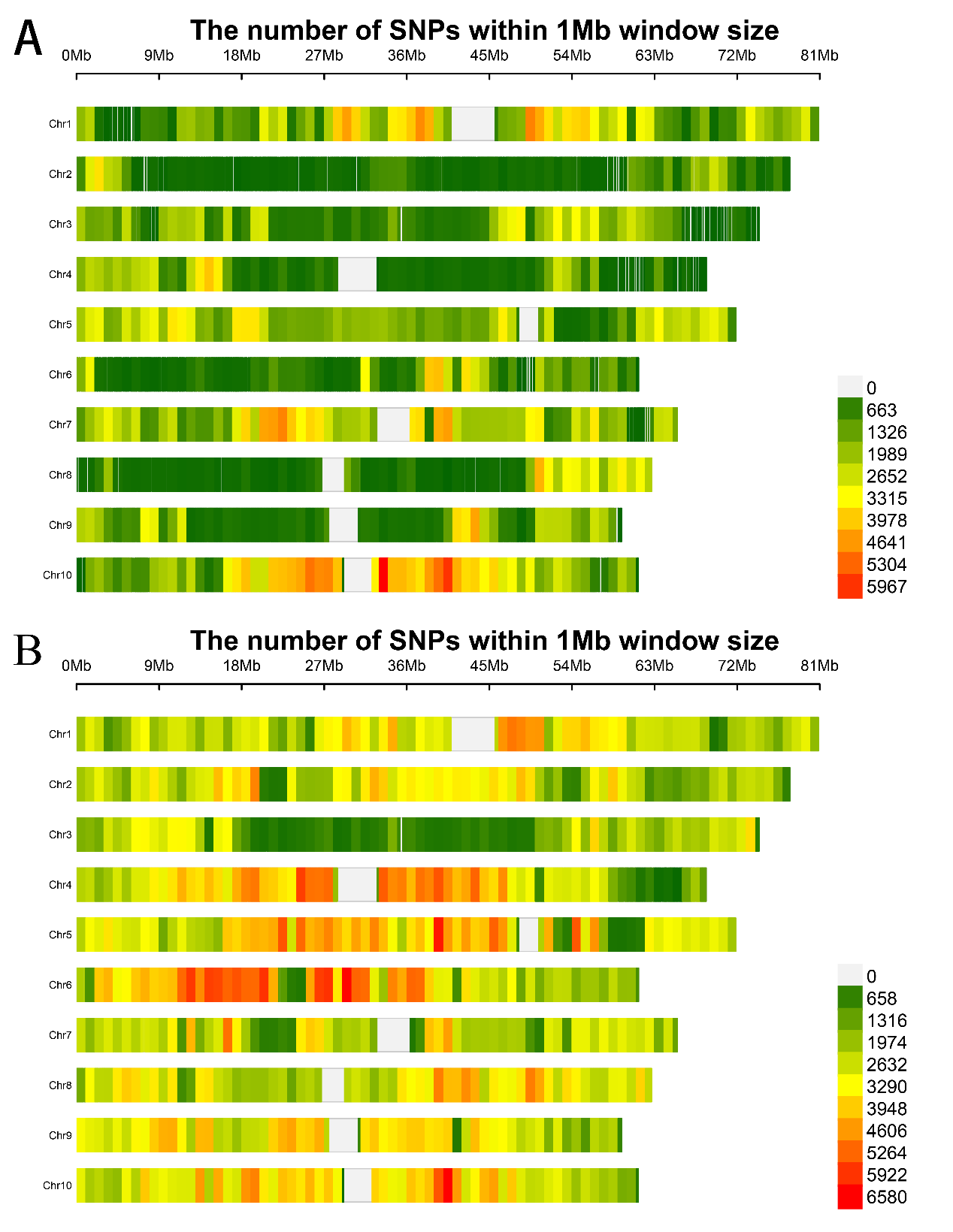
**

**Fig S1.** SNP marker density distribution

**A and B**, The distribution of SNP variations in 2 sorghum varieties on 10 chromosomes. A represents AJ2055, and B represents RN133.


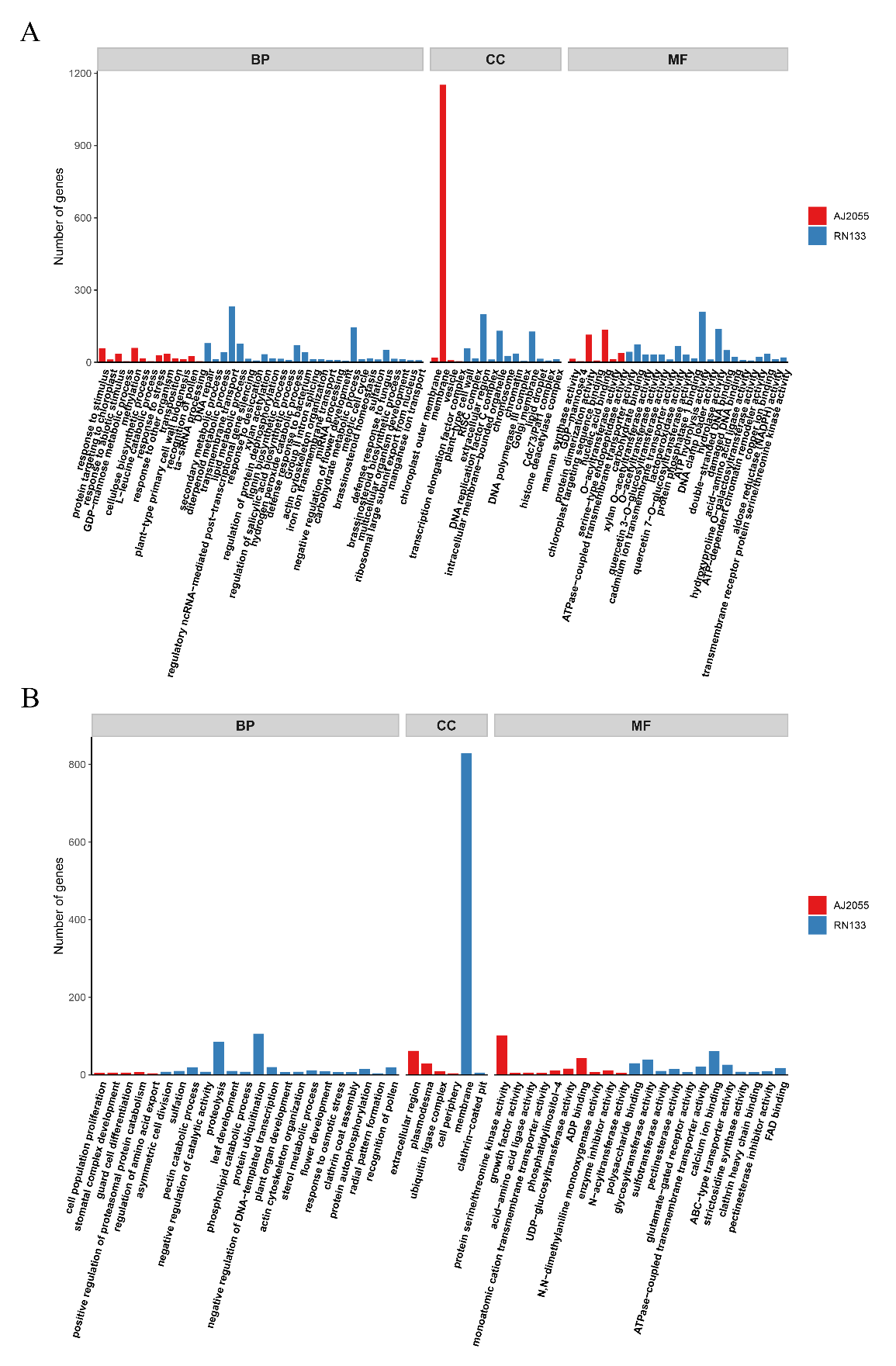
**Fig S2.** Go enrichment analysis.

**A and B**, Functional enrichment analysis of unique SNPs and InDels variants in two sorghum varieties (*P*<0.05). Red represents AJ2055, and blue represents RN133. The analysis includes three categories: Biological Process (BP), Cellular Component (CC), and Molecular Function (MF).


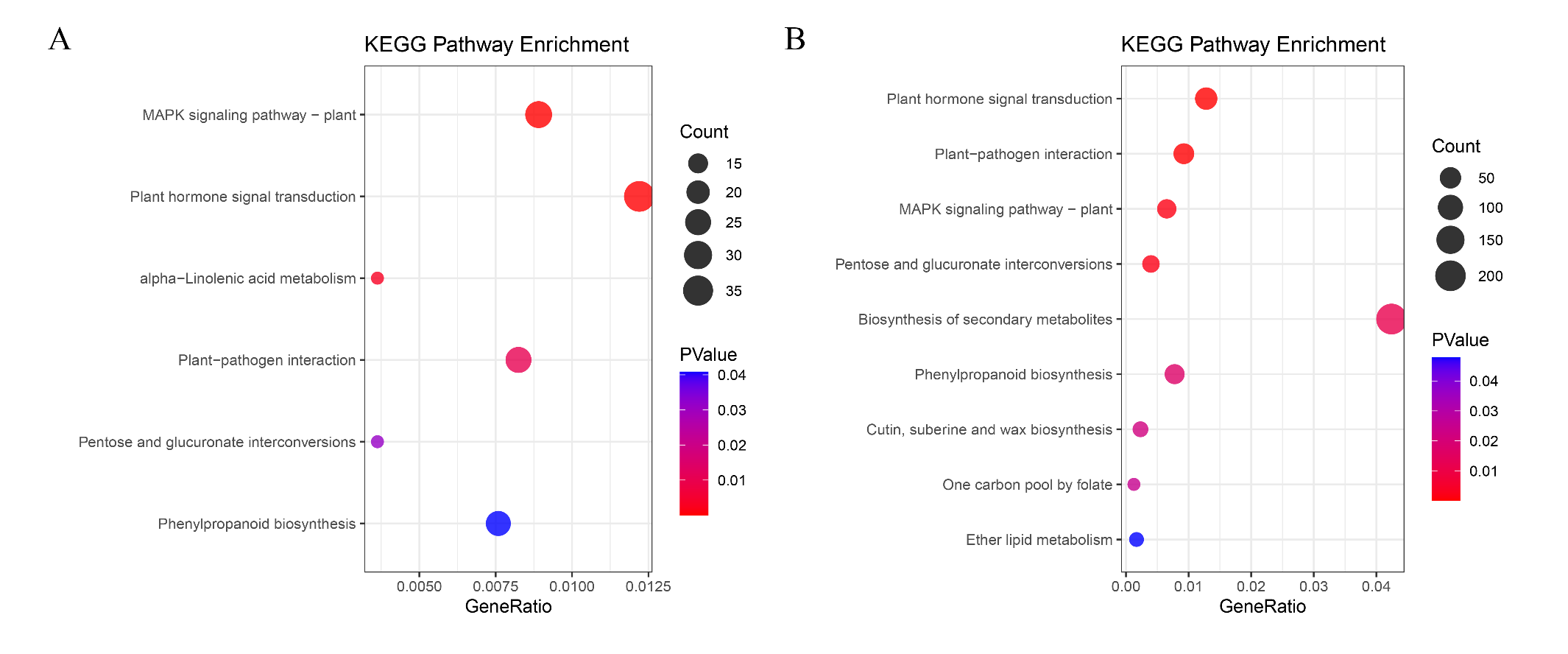


**Fig S3.** Significant enrichment pathway of InDels variants.

**A and B**, KEGG enrichment analysis of InDels variants in two sorghum varieties (*P*<0.05). A represents AJ2055, and B represents RN133.


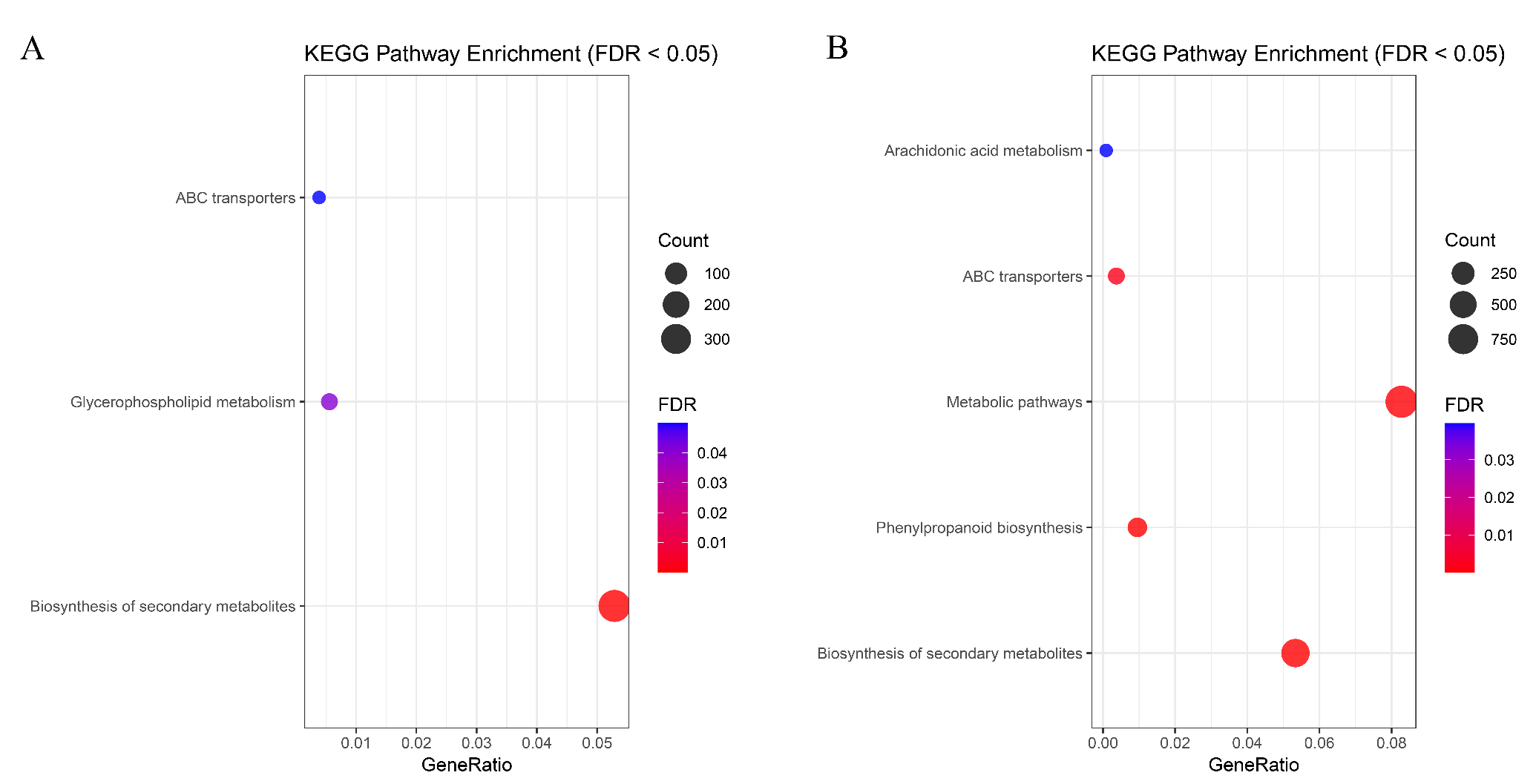


**Fig S4.** Significant enrichment pathway of SNPs variants.

**A and B,** KEGG enrichment analysis of SNPs variants in two sorghum varieties (FDR<0.05). A represents AJ2055, and B represents RN133.

**Fig S5.** Significant enrichment pathway of InDels variants.

**A and B,** KEGG enrichment analysis of InDels variants in two sorghum varieties (FDR<0.05). A represents AJ2055, and B represents RN133.
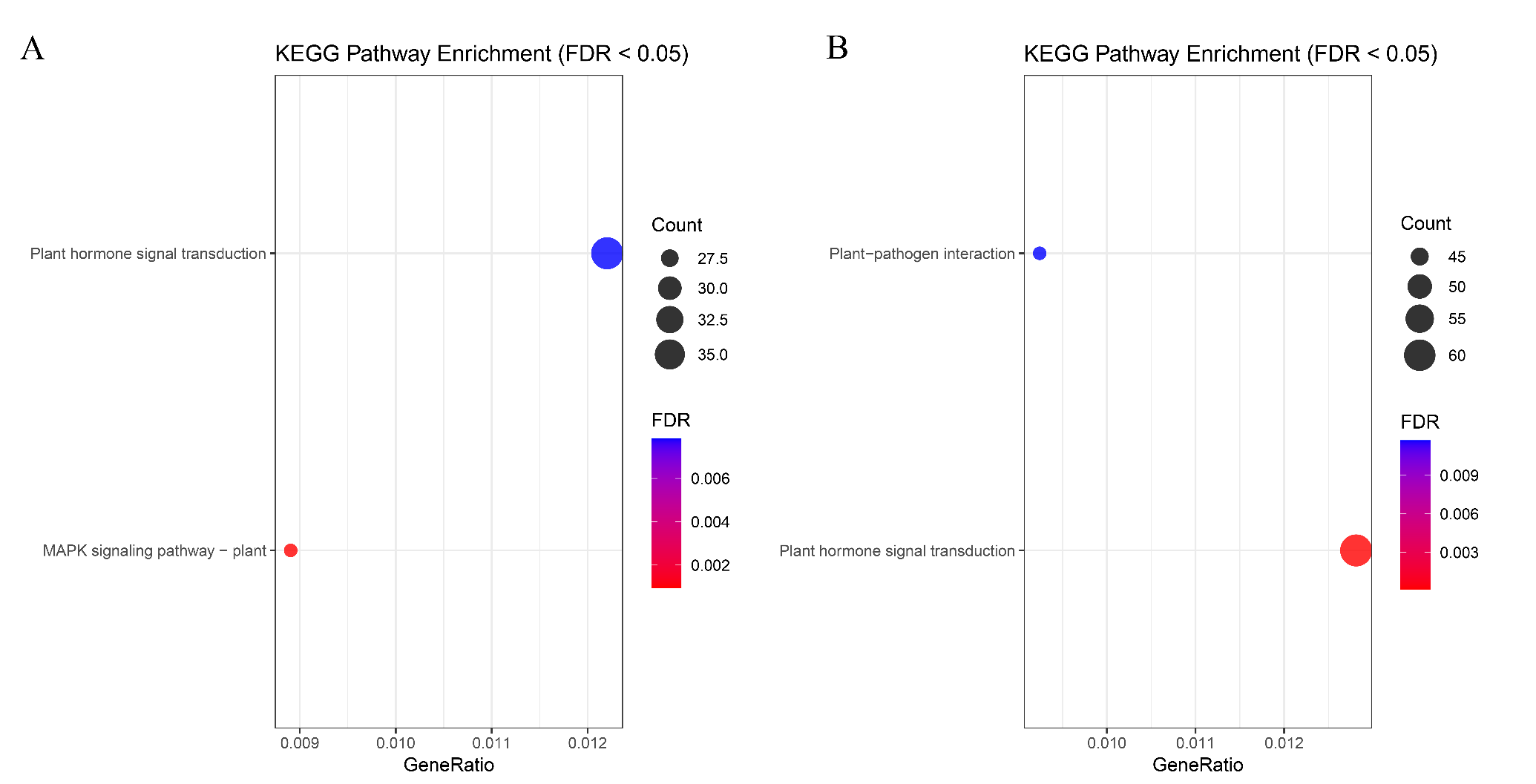


**Table S1.** Statistics of InDels variant gene regions and mutation types

| Name | Upstream | Stop gain | Stop loss | Frameshift deletion | Frameshift insertion | Non-frameshift deletion | Non-frameshift insertion | Intronic | Downstream | Heterogenous rate | Total |
| --- | --- | --- | --- | --- | --- | --- | --- | --- | --- | --- | --- |
| B2055 | 17964 | 30 | 0 | 540 | 435 | 1689 | 1597 | 22879 | 13124 | 0.03 | 177391 |
| N133 | 28884 | 55 | 4 | 808 | 723 | 2616 | 2612 | 37343 | 21435 | 0.041 | 296856 |
